# Grid-wise simulation acceleration of the electromagnetic fields of 2D optical devices using super-resolution

**DOI:** 10.1038/s41598-023-27449-y

**Published:** 2023-03-06

**Authors:** Jangwon Seo, Insoo Kim, Junhee Seok

**Affiliations:** School of Electrical Engineering, Korea University, Seoul, Korea

**Keywords:** Mathematics and computing, Computational science, Optical materials and structures

## Abstract

The significance of simulation has been increasing in device design due to the cost of real test. The accuracy of the simulation increases as the resolution of the simulation increases. However, the high-resolution simulation is not suited for actual device design because the amount of computing exponentially increases as the resolution increases. In this study, we introduce a model that predicts high-resolution outcomes using low-resolution calculated values which successfully achieves high simulation accuracy with low computational cost. The fast residual learning super-resolution (FRSR) convolutional network model is a model that we introduced that can simulate electromagnetic fields of optical. Our model achieved high accuracy when using the super-resolution technique on a 2D slit array under specific circumstances and achieved an approximately 18 times faster execution time than the simulator. To reduce the model training time and enhance performance, the proposed model shows the best accuracy (R2: 0.9941) by restoring high-resolution images using residual learning and a post-upsampling method to reduce computation. It has the shortest training time among the models that use super-resolution (7000 s). This model addresses the issue of temporal limitations of high-resolution simulations of device module characteristics.

## Introduction

Because the current device module design was built using the expertise and knowledge of professionals, it is difficult to deviate from the conventional design. Furthermore, finding a suitable device design is a costly and time-consuming task, even for the experts. Consequently, to assist the experts in finding the device design, research is being conducted on a simulator that rapidly and precisely determines the features of the device designs by solving partial differential equations^1,2^. For example, a Matlab-based package simulator called Maxwell’s equations by the finite-difference frequency-domain (MaxwellFDFD) has been developed to solve Maxwell’s equations for various materials and boundary conditions in the frequency domain^3^. Using the simulator, various characteristics of the input device design, such as power, an electric field, and a magnetic field, can be obtained through simulation calculations. When using the simulator to conduct an iterative device design process, the outcomes of various structures may be accumulated, resulting in the formation of big data.

Most of the currently used simulators, including the classic simulator, integrate the calculations from the input space’s fine grid to solve the partial differential equations, such as Maxwell’s equations derived from the input space. Thus, the accuracy of the approximated solution highly depends on the size of the split fine grid. Consequently, increasing the number of fine grids reduces the size of the measurement unit, improving the accuracy of the calculations’ outcome while exponentially increasing the calculation time.

Deep and machine learning, which have been rapidly evolving recently, are being used in various fields such as image and natural language processing to effectively identify the relationship between complex input and output. Additionally, machine learning has broadened its application to address issues with the current device module simulator^4–7^. Recent studies have shown that advances in machine learning models, such as deep neural networks, mostly focus on solutions for partial differential equations, such as Maxwell’s equations for calculating electromagnetic values^8–13^ and there are few studies on grid-wise techniques. In this study, we found that the input space simulator’s calculation results may be seen as an image. The approximated calculation results of the input spaces’ coarsely split grid correspond to the image’s pixel values. This study used image super-resolution, which is used for image processing, to reduce the simulator’s computation time while maintaining the accuracy. Super-resolution is a technology that converts a low-resolution image into a high-resolution image^14–21^. To train the super-resolution model, both low-resolution and high-resolution images are required. In this study, we modified the coarse grid’s size in the given input space to modulate the images’ resolution. The high-resolution data were created by calculating the simulator with many coarse grids of relatively small size, whereas the low-resolution data were created using a few coarse grids of a relatively large size.

In this study, we propose a fast residual learning super-resolution (FRSR) convolutional network, a model that reconstructs high-resolution simulation data from low-resolution simulation data using a super-resolution method. The experimental results aimed to address the issue of temporal simulator limitations with high model accuracy and short execution time.

## Results and discussion

In this section, we outline the experimental findings of our proposed approach. Briefly, our proposed method effectively reduced the computing time while maintaining comparable accuracy of the calculation result. Additionally, using the residual learning and post-upsampling method, our proposed method reduced the number of parameters and enhanced the performance compared with the conventional methods implemented in an image super-resolution problem. The detailed experimental results are demonstrated in subsequent sub-sections.

### Model evaluation

The experiment used the MaxwellFDFD simulator to determine the optical device parameters, such as power, an electric field, and a magnetic field, according to the given input space. The calculated electric and magnetic fields each consist of three vector planes, each of which partitions the same simulation space into a fine grid. Because the electric and magnetic fields are perpendicular to each other, there is a zero component between the two field’s components. Except for the zero component, the electric field has two components and the magnetic field has one component, which are expressed as ${E}_{x}$, ${E}_{y}$, and ${H}_{z}$, respectively. This study used images of three channels composed of ${E}_{x}$, ${E}_{y}$, and ${H}_{z}$.

A total of 10,800 pairs of data, each consisting of a low- and high-resolution image, were produced using a simulator. In the same simulation space, changing the coarse grid’s size modified the images’ resolution. Among 10,800 pairs of the generated data, 9600 were used to train the proposed model and 1200 were used to test the actual model’s performance. Seventy percent of the training set was used to train the model, whereas 30% was used as a validation set to tune the model’s hyper-parameter.

The proposed FRSR model reconstructs a high-resolution electromagnetic field image from a low-resolution image. The residual learning method was included in the proposed method because it has shown novel performance in image-related tasks^22,23^. Additionally, the post-upsampling technique was implemented to reduce the model’s parameter count, while maintaining model accuracy^24^. To investigate its efficiency, the proposed method was compared with the traditional methods implemented for the super-resolution problems, such as bicubic interpolation^25^, SRCNN^17^, FSRCNN^18^, VDSR^19^, and LapSRN^24^ models.

The proposed FRSR model outperformed the traditional methods in terms of reconstructing high-resolution simulation data. The FRSR model had an R^2^ score of 0.9912, which was the highest compared with the other models in the validation set. The RMSE value of the FRSR model was 0.0033 in the validation set, which was the highest compared with the other models. The detailed comparison results of FRSR and the traditional models are shown in Supplementary Tables S1 and S2. Generally, the models that had deep learning applied performed better across all axis than the interpolation model on the training and validation sets.

While our proposed model demonstrated efficacy in high-resolution data reconstruction, it also demonstrated a computational efficiency compared with conventional techniques. The training time for the FRSR model was 7000 s, which is approximately 3.4 times quicker than the VDSR model, which had equivalent prediction performance. Although the FRSR model’s size is considerably lighter than the VDSR model, the use of the post-upsampling and the residual module maintained its reconstruction performance. Table 1 shows the detailed comparison results of training time among the proposed and conventional deep learning-based models for super-resolution. Because the training time is short, it is more efficient to use the ensemble, and easy to apply it to the data with a wider dimension than the data used in this study. The final prediction result of FRSR is calculated using an ensemble of eight independent FRSR models based on the computational efficiency during the training step.

**Table 1:** Comparison of training time for each of the five models: SRCNN, FSRCNN, VDSR, LapSRN and FRSR on the 6720-training data set.

Model	SRCNN	FSRCNN	VDSR	LapSRN	FRSR
Training time (s)	13,361	8076	24,083	7524	7000

Among the super-resolution models, the FRSR model has the shortest training time.

In 1200 test set samples, the proposed FRSR model also showed best performance compared with the conventional approaches in terms of reconstructing the high-resolution simulation data. The R^2^ score value of the FRSR model was 0.9941 in the test set, which was the highest among the analyzed models. Additionally, the FRSR model’s RMSE value was 0.0028 in the test set, which was the highest of the examined models. The detailed comparison results of the FRSR and traditional models are shown in Supplementary Tables S3 and S4.

There are quantitative evaluation metrics used in many studies to evaluate the quality of Super-resolution results. The evaluation scales applied in this study are Peak Signal-to-Noise Ratio (PSNR), Structural Similarity Index (SSIM), Visual Information Fidelity (VIF), Universal Quality Image Index (UQI), Relative Average Spectral Error (RASE), Spectral Angle Mapper (SAM) and Spatial Correlation Coefficient (SCC). In Supplementary Table S5, the FRSR model shows similar or better performance in other indicators except for the VIF evaluation metric.

**Figure 1:**
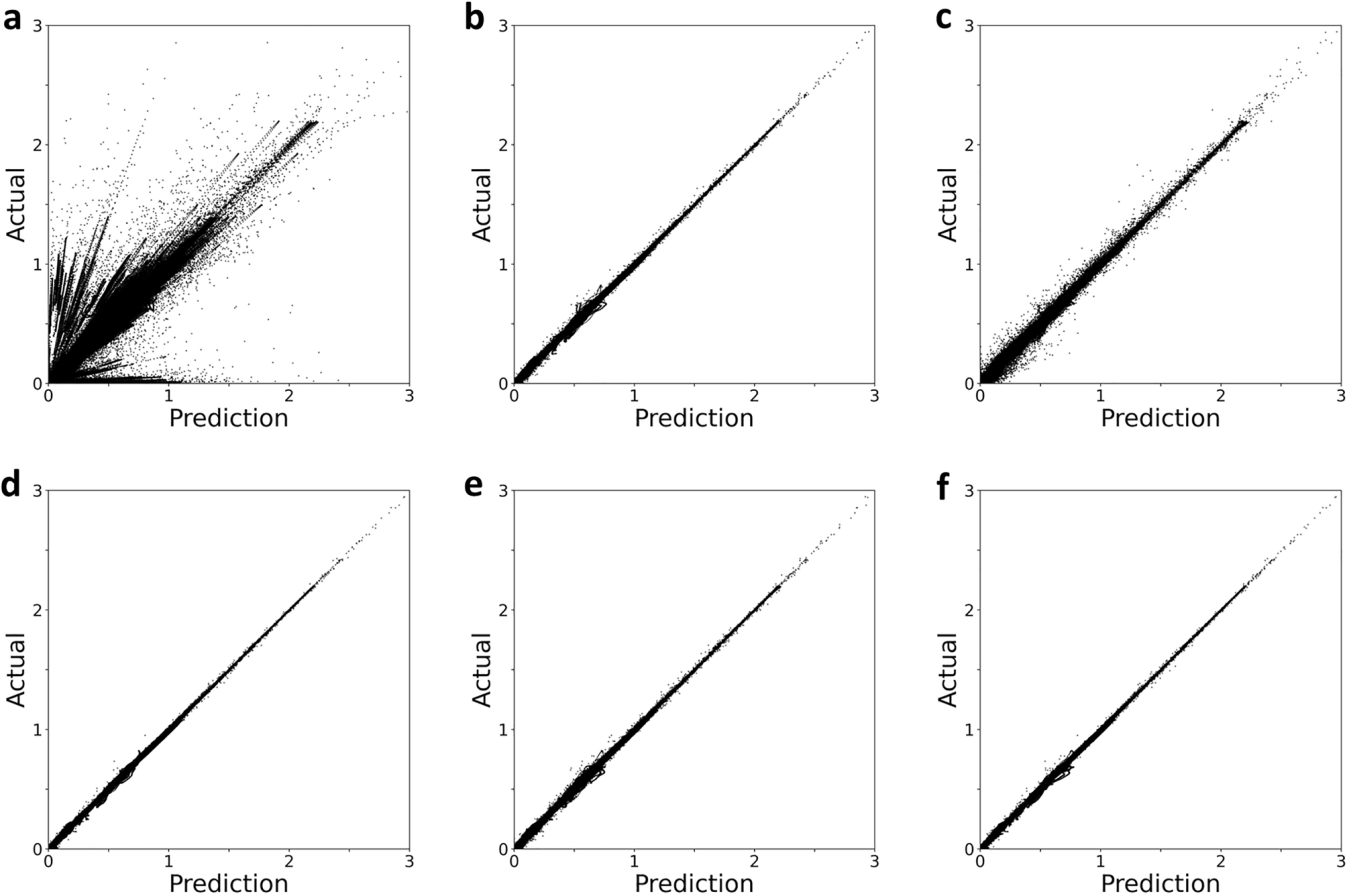
Model performance results for six models in the test set are expressed as scatter plots of model-predicted and actual values. (**a**) scatter plot when bicubic interpolation is applied, (**b**) SRCNN is applied, (**c**) FSRCNN is applied, (**d**) VDSR is applied, (**e**) LapSRN is applied, and (**f**) FRSR is applied. The points represent E_x_, E_y_ and H_z_.

Figure 1 shows the actual and predicted values for three channels of 1,200 test set samples as one plane of a scatter plot when the electromagnetic field magnitude is predicted using the trained model. Figure 1a shows a model to which bicubic interpolation is applied, and Fig. 1b–f present the models to which SRCNN, FSRCNN, VDSR, LapSRN, and FRSR are applied to, respectively. Among the six models, our proposed FRSR performed the best in the test set prediction. Compared with the interpolation model, the deep learning-based model also performed better in the test set prediction. Owing to the characteristic of a bicubic interpolation that tends to fill in the zero values of a boundary space into an interpolated real value, many outliers far from the trend line were produced in the case of the interpolation method in Fig. 1a. Additionally, a rectified linear unit function (ReLU) was applied to the predicted value because the electromagnetic field’s magnitude cannot be negative. The points are distributed on the $\mathrm{x}$ and $\mathrm{y}$-axis of the scatter plot if the predicted value is negative, which is calculated as a value of zero.

**Figure 2:**
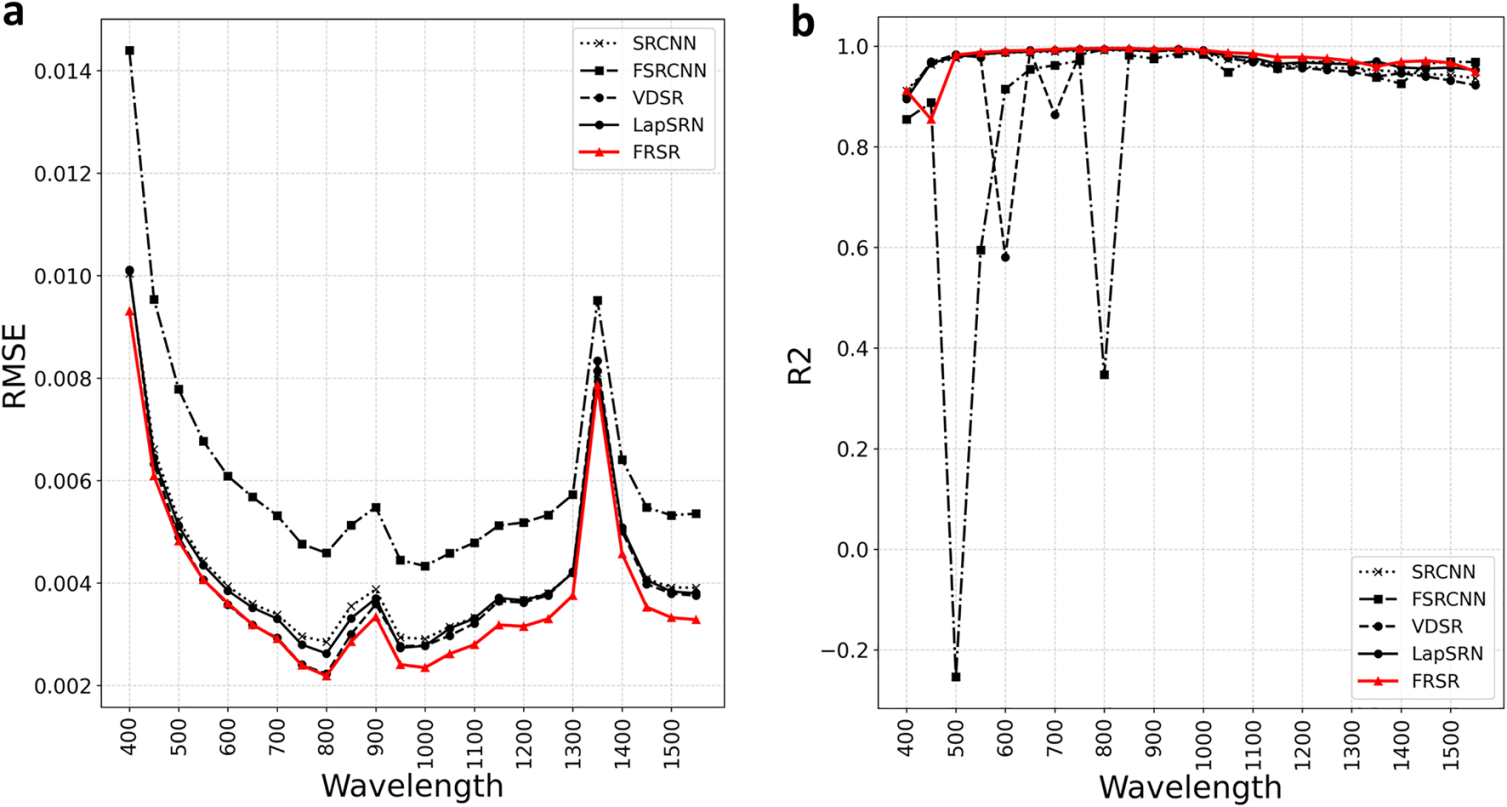
Comparison graph of RMSE and R2 scores according to the wavelength of the five models: SRCNN, FSRCNN, VDSR, LapSRN and FRSR in the test set divided into 24 wavelengths. (**a**) The graph evaluating each model with RMSE and (**b**) the graph evaluating each model with R2.

The prediction results of the proposed and conventional models in 24 various wavelengths are shown in Fig. 2 for a detailed illustration of the novelty of the proposed FRSR model. Figure 2 shows the comparison of the RMSE and R^2^ scores according to the wavelengths of the five models: SRCNN, FSRCNN, VDSR, LapSRN, and FRSR, in a test set divided into 24 wavelengths. Each wavelength has 500 data sets. The FRSR model also performed well, with an average RMSE score of 0.0038. Figure 2b shows the values that the FSRCNN model cannot predict for a specific wavelength. The FRSR model performs consistently for all wavelengths and offers the best performance, with an average R^2^ score of 0.9738, as shown in Table 2. Each model performed worse when using the dataset according to the wavelength than when using the entire data set. Although the VDSR’s performance was high using the entire dataset, it showed worse performance than the SRCNN when analyzed with datasets according to the wavelength. The proposed FRSR performed well on both the full and wavelength-specific datasets.

**Table 2:** Comparison results of average RMSE and average R^2^ scores according to the wavelength of five models, SRCNN, FSRCNN, VDSR, LapSRN, and FRSR, in the test set divided into 24 wavelengths.

Wavelength data	SRCNN	FSRCNN	VDSR	LapSRN	FRSR
Average of RMSE	0.0043 $\pm$ 0.0017	0.0061 $\pm$ 0.0022	0.0041 $\pm$ 0.0018	0.0042 $\pm$ 0.0019	0.0038 $\pm$ 0.0017
Average of R^2^	0.9684 $\pm$ 0.0214	0.8628 $\pm$ 0.2722	0.9435 $\pm$ 0.0820	0.9733 $\pm$ 0.0319	0.9738 $\pm$ 0.0310

Among the super-resolution models, the FRSR model demonstrates the best performance.

### Execution time

In the classic simulator, the Maxwell equation is solved to determine the magnitude of the electromagnetic field. The simulation took 180 s to complete, 170 s to generate high-resolution simulation data and 10 s to generate low-resolution simulation data. The generation time of low-resolution simulation data and the computation time of reconstructed high-resolution data are combined to determine the overall execution time of the proposed FRSR model. The proposed FRSR and conventional super-resolution models generated the entire dataset of 9600 pairings in approximately 27 h, which is approximately 18 times faster than the simulator, which required 480 h. The FRSR model’s execution time was 26.93 h, and Table 3 analyzes the comparative results.

**Table 3:** Comparison of average execution time between a MaxwellFDFD simulator and super-resolution model.

# of samples	MaxewllFDFD	SRCNN	FSRCNN	VDSR	LapSRN	FRSR
1	180 s	10.11 s	10.09 s	10.11 s	10.12 s	10.10 s
1200	3600 m	202.1 m	201.8 m	202.3 m	202.4 m	201.95 m
9600	480 h	26.95 h	26.91 h	26.97 h	26.98 h	26.93 h

One sample means a pair of high-resolution and low-resolution data. When super-resolution is applied, it is approximately 18 times faster than the simulator for all samples. Among the super-resolution models, the FRSR model has the second shortest execution time.

### Examples

To verify the validity of the proposed FRSR model, additional experiments were performed to restore a low-resolution image to a high-resolution image.

Supplementary Fig. S1 shows an example of a study showing the RMSE error between the predicted and actual values of the ${E}_{x}$ component for a specific slit design at 800 nm wavelength as a heatmap. Supplementary Figure S1 shows the exact slit design used the shape shown in Supplementary Fig. S1a and show the error when the low-resolution image is restored to the high-resolution image using the interpolation, SRCNN, FSRCNN, VDSR, LapSRN, and FRSR models, respectively. Small errors are shown in black, whereas large errors are highlighted in white. The figures demonstrate that the obstacle border section and simulation edge region have poor prediction ability. Because the magnitude of the electromagnetic field fluctuates significantly, regarding the obstacle boundary prediction, it is more difficult to predict than in other regions. Additionally, when deep learning is used, the simulation’s edge part performs poorly in the prediction because zero padding is performed. In Supplementary Fig. S1e,g, there are fewer white areas to which VDSR and FRSR were applied than other models. The power flux, a physical property, can be calculated through the electromagnetic field predicted by the model. Calculate the power flux by calculating the Poynting’s vector, defined as $\mathrm{S}\left(\mathrm{r}\right)=\mathrm{E}(\mathrm{r})\times \mathrm{H}(\mathrm{r})$, where $\mathrm{E}(\mathrm{r})$ and $\mathrm{H}(\mathrm{r})$ denote the electric and magnetic fields at point $\mathrm{r}$. It was applied in the test set, and the power flux was calculated on the upper observation plane of the edge with a large error with zero padding. In Supplementary Table S6, a comparison between the power flux calculated through the simulator and predicted through the deep learning model shows an R^2^ score of over 0.98 after super-resolution.

**Figure 3:**
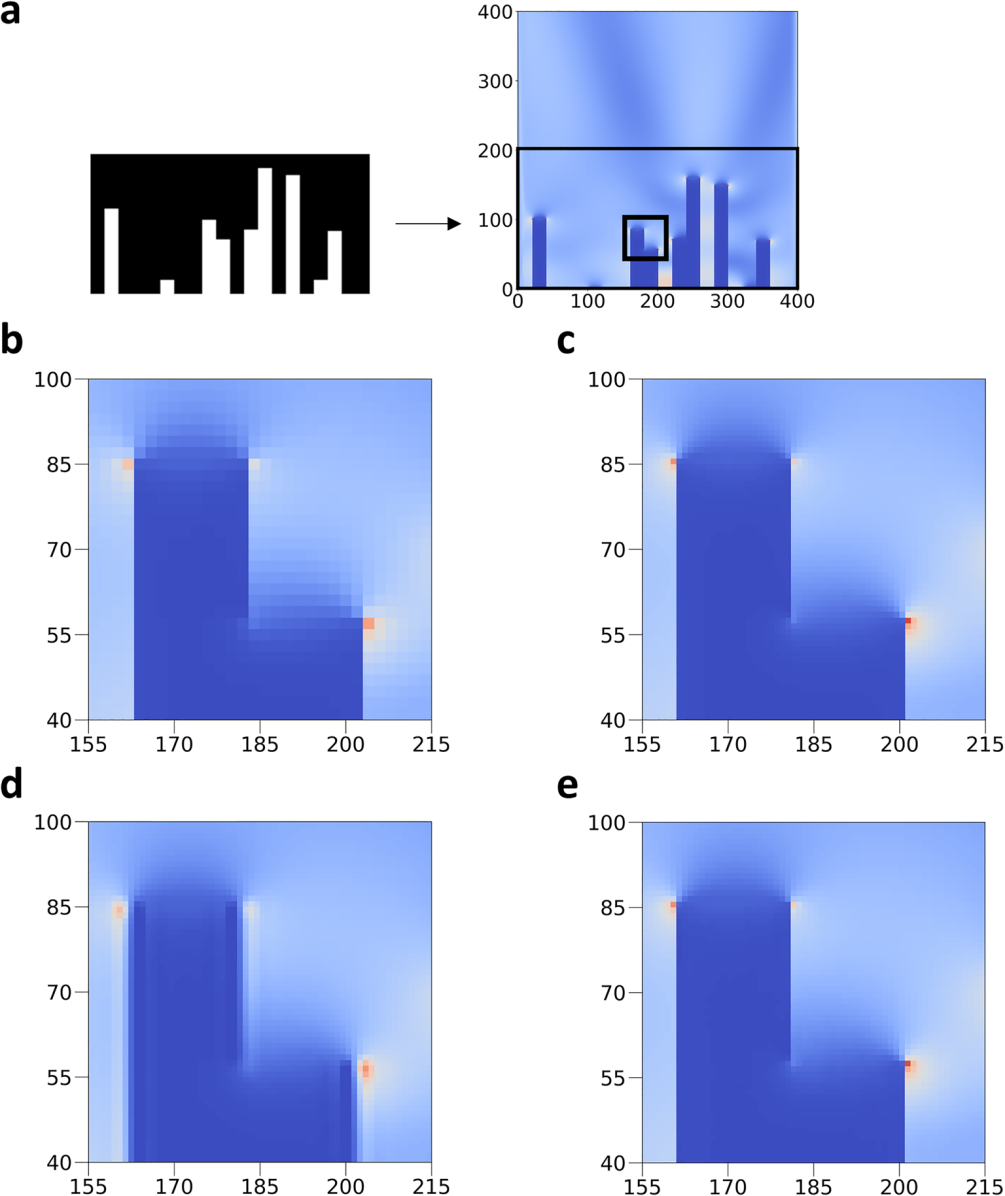
An example of a study showing the E_x_ component of the electromagnetic field magnitude image for a specific slit design at 800 nm wavelength as a heatmap. (**a**) Device design is used in the example. (**b–e**) are enlarged images of a specific part in (**a**). (**b**) Low-resolution image. (**c**) High-resolution image. (**d**) An image is restored to a high resolution by applying interpolation to the low-resolution image. (**e**) An image is restored to a high resolution by applying FRSR to a low-resolution image.

Figure 3a shows a simulation-based device design under the assumption that a plane wave of 800 nm is generated in the 2000 $\times$ 3600 nm optical device simulation space. The ${E}_{x}$ component of 2000 nm $\times$ 2000 nm, the region including the device design, was shown as a heatmap of a high-resolution (400 $\times$ 400) image with a coarse cell size of five. Specific parts of the simulation space image of the ${E}_{x}$ component of Fig. 3a are shown in Fig. 3b–e. Figure 3b shows a low-resolution image, Fig. 3c shows a high-resolution image, Fig. 3d shows a high-resolution image obtained by reconstructing the low-resolution image of Fig. 3b through the interpolation model, and Fig. 3e shows a high-resolution image obtained by reconstructing the low-resolution image of Fig. 3b through the FRSR model. Figure 3d and e, are restored similarly to the high-resolution images in Fig. 3c. However, in Fig. 3d, unlike the high-resolution image in Fig. 3c, the end of the obstacle cannot be properly restored, and the crushed area is visible; but in the FRSR model in Fig. 3e, it was similarly restored.

## Conclusion

The conventional simulator generates various device design parameters, including the electric and magnetic field’s data discussed in this study. To address the limitations of the current simulators, we propose the FRSR model, which is suitable for reconstructing high-resolution simulation data from low-resolution simulation data. The proposed model outperformed conventional super-resolution models in terms of reconstruction performance while reducing the training time. The novel performance of the proposed model is attributed to the implementation of the residual module and the post-up sampling technique. Additionally, the proposed model successfully accelerated the computation time of the high-resolution data by 18 times compared with the conventional simulator.

The image resolution is increased using super-resolution models of images. Because both the device module in the image and the simulator consist of high-order numbers, their characteristics are similar. This study’s attempts to increase the resolution of the simulation data using the super-resolution models demonstrated novel performance. Consequently, our study suggests one possible solution to the temporal limitations of the device module simulators. We expect this research to be useful for engineers and researchers in the field of devices.

This study successfully performed twofold upsampling in this paper by applying the super-resolution technique. Although upsampling has been successfully applied up to 2 times based on research, the goal of future research will be to use higher upsampling by learning various scale factors to increase the versatility of the proposed model. In the super-resolution learning method of this study, learning was performed by defining the size of each vector component as one channel size. The interaction of electric and magnetic fields causes electromagnetic waves. The current model applies only the vector magnitude, but the model can be further improved by additional training of the electromagnetic field vector direction and physical properties (e.g., transmittance) to consider the relationship between vectors.

## Methods and materials

By implementing a super-resolution model, our proposed method predicts the device characteristic values of the electric and magnetic fields calculated using a simulator from the designed shape of the material. This section demonstrates the overall experimental settings used in this study, and the detailed FRSR model structure.

### Generating dataset

**Figure 4:**
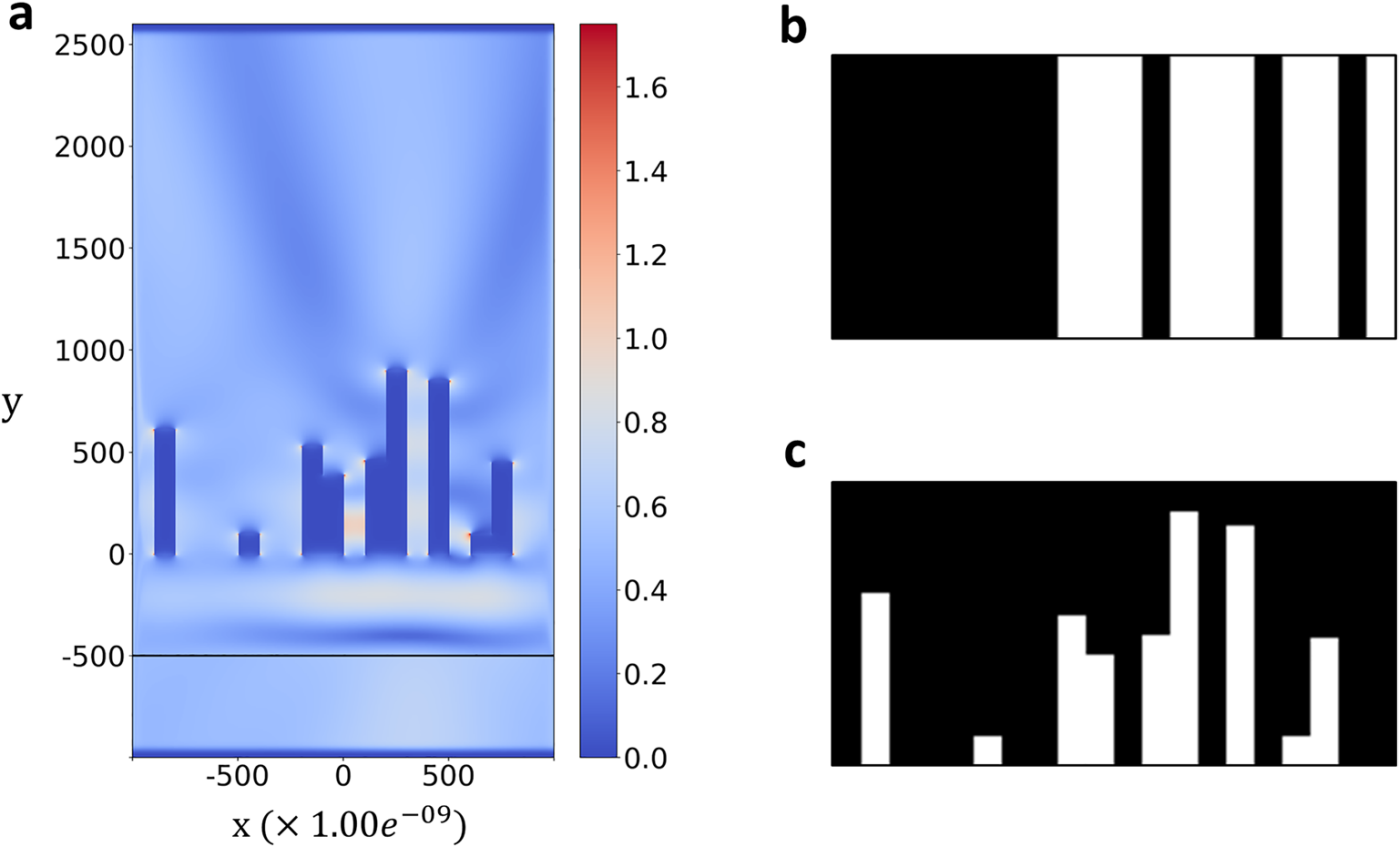
Examples of MaxwellFDFD simulation space and device design examples. (**a**) generates a plane wave of 800 nm at y =  − 500 and passes through a randomly designed image between y = 0 and y = 1000. (**b**) is simple random data, and (**c**) is complicated random data. The white parts of the images represent the object material of the simulation, and the black parts represent vacancies.

The simulation conditions for generating data and the slit design fabrication method were conducted under the same conditions as in the study(Simulation acceleration for transmittance of electromagnetic waves in 2D slit arrays using deep learning)^8^. In this simulation, the 2000 $\times$ 3600 nm optical device module is vacuum-injected with plane waves, as shown in Fig. 4a. A total of 24 plane waves that were injected at $\mathrm{y}=-500$ at intervals of 50 nm from 400 to 1550 nm were used for the simulation. Various characteristics, such as electric field, magnetic field, and power, are calculated as the plane wave passes through the device design. A detailed explanation of the device design is shown in Fig. 4b and c. The generated device design is used to compute the electromagnetic field of an input space. Figure 4a shows the visualization results of the simulation. A vacuum was used as the simulator’s background material, while silver was used for the traditional simulator. Because of the simulation, the values of the electric and magnetic fields can be obtained by solving the Maxwell equations. The Maxwell equation is written as follows:

1 $$\nabla \times E\left(r\right)= -i\omega \mu \left(r, \omega \right)H\left(r\right)-M\left(r\right),$$

2 $$\nabla \times H\left(r\right)=i\omega \varepsilon \left(r, \omega \right)E\left(r\right)+J\left(r\right),$$

where $E\left(r\right)$ and $H\left(r\right)$ represent the electric and magnetic fields at a given point $r$. $J\left(r\right)$, $M\left(r\right)$ represent the densities of the electric and magnetic fields at a given point $r$. $\varepsilon \left(r, \omega \right)$, and $\mu \left(r, \omega \right)$ represent permittivity and permeability. Equation (1) is Faraday's law, in which an electric field perpendicular to the magnetic field is generated by a changing magnetic field. Equation (2) is Ampere's law, in which a magnetic field perpendicular to the electric field is generated by a changing electric field. $E\left(r\right)$ and $H\left(r\right)$ are calculated from the equation.

The traditional simulations return E-field and H-field solutions of Maxwell’s equations, among other properties. E-field and H-field are coarse grids of length three each. Each coarse grid has three arrays composed of a set of vectors, and the array of each coarse grid is defined as an $\mathrm{x}$, $\mathrm{y}$, and $\mathrm{z}$ component. The E-field component arrays were named ${E}_{x}$, ${E}_{y}$, and ${E}_{z}$, whereas the H-field component arrays were named ${H}_{x}$, ${H}_{y}$, and ${H}_{z}$. The electromagnetic field resulting from the simulation can be expressed as six components ${E}_{x}$, ${E}_{y}$, ${E}_{z}$, ${H}_{x}$, ${H}_{y}$, and ${H}_{z}$, and each component is composed of a set of vectors. Because the electric and magnetic fields are perpendicular to each other, and the direction of the electromagnetic wave propagation in the simulator is set to a direction that can penetrate the device design from the wave source, the magnitudes of ${E}_{z}$, ${H}_{x}$, and ${H}_{y}$ components are set to zero. As a result of analyzing the six components calculated through simulation; therefore, the E-field and H-field are composed of three components,${E}_{x}$, ${E}_{y}$ and ${H}_{z}$. In this study, it was defined as an image with three channels composed of two-dimensional components ${E}_{x}$, ${E}_{y}$ and ${H}_{z}$.

In the entire simulation space, only the square-shaped simulation space of 2000 $\times$ 2000 nm between $\mathrm{y}=0$ and $\mathrm{y}=2000,$ including the device design part, is used. Divide the simulation space into multiple grids and calculate the properties of each grid. The unit of fine grid that may be configured as the simulator’s condition is divided into squares of 10 nm and 5 nm to calculate the device characteristics according to the resolution. A 10-nm fine grid is 4 times wider than a 5-nm fine grid, and four characteristic values calculated from a 5-nm fine grid are expressed as a single value. That is, the larger the fine grid size of the simulator, the lower the resolution, and the smaller the fine grid size, the higher the resolution.

To apply the three components ${E}_{x}$, ${E}_{y}$ and ${H}_{z}$ to super-resolution, the vector values of each component are converted into magnitude. For various device designs, the size of the electric and magnetic fields of each coarse grid was calculated using a simulator to compose 10,800 device module characteristic data for each resolution. From the total amount of data, 1200 were used as a test set, 6720 were used for training, and 2880 were used as a validation set to validate the trained data. One sample consists of a low-resolution image and two high-resolution images.

### Training model

To restore a low-resolution image to a high-resolution image, six models were applied and compared in this study. The interpolation method using simple calculations and the SRCNN, FSRCNN, VDSR, LapSRN, and FRSR methods using deep learning were used. The interpolation block was applied as the up-sampling method of the SRCNN, VDSR, and FRSR super-resolution models, and the detailed structure can be found in Supplementary Table S7. ReLU was applied to the result value to output the final reconstructed image because the result value predicted through the model cannot be negative.

**Figure 5:**
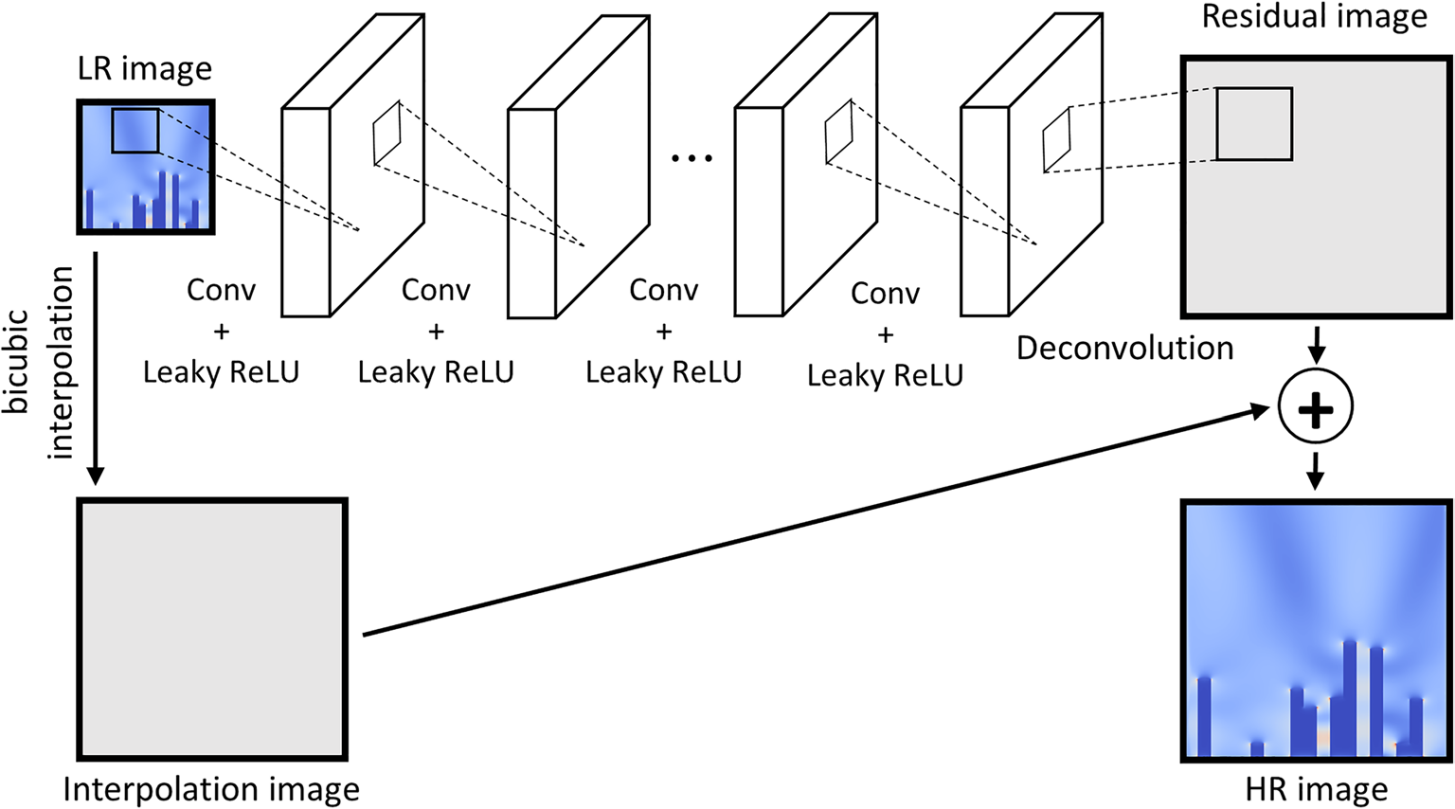
FRSR model architecture of the super-resolution model. We applied nine convolutional layers, and the last layer is the deconvolution layer. Leaky-ReLU was applied as an activation function between the eight layers except for the deconvolution layer. The residual image generated through the convolutional layer is combined with the interpolation image generated through bicubic interpolation to restore a high-resolution image.

Figure 5 depicts the structure of the FRSR model proposed in this study. The post-upsampling method is used to include the low-resolution image directly into the model. The detailed network architecture for the FRSR model is provided in Supplementary Table S8. Leaky-ReLU (alpha = 0.3) was used for the activation function, and the padding was performed using the same size. Leaky-ReLU was used because learning may not occur even if the value of the next layer is positive if the differential value of all coarse grids in one layer is zero. When restoring a high-resolution image, bicubic interpolation is used to merge the interpolation image with the residual image generated after the convolutional layers have been applied to the low-resolution image.

3 $$F\left(X\right)= \mathrm{H}\left(\mathrm{X}\right)- {X}_{identity}.$$

4 $$F\left(X\right)= \mathrm{H}\left(\mathrm{X}\right)- {X}_{interpolation}.$$

It is assumed that $\mathrm{X}$ serves as the input for the layers and that $\mathrm{H}\left(\mathrm{X}\right),$ the layer’s output, has the same dimension as $\mathrm{X}$. Because FRSR is a post-upsampling model, ${X}_{identity}$ returns the image obtained by interpolating $\mathrm{X}$. ResNet trains a residual function as opposed to a general neural network that trains $\mathrm{H}(\mathrm{X})$.

The SRCNN model is a pre-upsampling method that uses bicubic interpolation to preprocess a low-resolution image to make it the same size as a high-resolution image. The detailed network architecture for the SRCNN model is provided in Supplementary Table S9. The converted image passes through three layers to restore the high resolution. ReLU and Linear were used for the activation function, and the padding was performed using the same size.

The FSRCNN is a model developed to quickly train SRCNN. The difference between SRCNN and FSRCNN is that SRCNN upsamples in the deconvolution stage using the post-upsampling method. FSRCNN consists of eight layers. The detailed network architecture of the SRCNN model is provided in Supplementary Table S10. The deconvolution layer scales the stride and transforms it into a high-resolution image. For each layer, the activation function used parametric rectified linear unit function (PReLU), and the padding was performed using the same size.

There are 20 convolutional layers in the VDSR model, which is used in image processing. However, in the case of the optical device data used in this study, it takes a very long time to learn when all 20 layers are used. Because prediction performance is important, but execution time is also significant, simulation is used in the VDSR model by reducing the number of layers to nine. The pre-upsampling method and residual method were applied in the same way as in VDSR. The detailed network architecture for the VDSR model is provided in Supplementary Table S11. Bicubic interpolation was used to convert the low-resolution image to the size of the high-resolution image, and this image is used as the model's input. ReLU was applied to the activation function used between each layer, and the padding was performed using the same size.

The LapSRN model consists of an embedding block, an upsampling block, and a residual block. The LapSRN model used a post-upsampling technique to reduce parameters and training time while maintaining model accuracy. The detailed network architecture for the LapSRN model is provided in Supplementary Table S12. The output of the embedding block was used as the input of the residual learning block, and the deconvolution layer was applied to make the image the same size as the target image. For each layer, the activation function used Leaky-ReLU applied, and the padding was performed using the same size.

The bicubic interpolation algorithm calculates using the product of pixel values of 16 adjacent pixels and weights according to distance. The bicubic interpolation equation is written as follows:

5 $$f\left(x,y\right)= \sum_{i=0}^{3}\sum_{j=0}^{3}{a}_{ij}{x}^{i}{y}^{j}$$

It takes 16 adjacent pixel values to determine one value.

All five deep learning models have a learning rate of 0.0003, the optimizer is Adam, and the loss is MSE. The kernel initializer of all convolution layers applied He Normal Initialization. For each super-resolution model, eight models were trained using an ensemble method, with an epoch of 150 and a batch size of 16. The data generation and training of the proposed super-resolution model applied in this study used an NVIDIA GTX 3090 and Ryzen 3600.

## Supplementary Information

Supplementary Information.

## Data Availability

The dataset and source codes for this work are publicly available at https://github.com/Jangwon37/maxwellfdfd-SR.
