# Supplementary material for: Grid-wise simulation acceleration of the electromagnetic fields of 2D optical devices using super-resolution
Source: Sci Rep. 2023 Mar 6;13:435. doi: 10.1038/s41598-023-27449-y (PMC9988857; doi:10.1038/s41598-023-27449-y)
Supplement: Supplementary file 1 — Supplementary Information. [file 41598_2023_27449_MOESM1_ESM.pdf]

Supplementary Materials

Grid-wise simulation acceleration of the electromagnetic fields of 2D optical devices using super-resolution

Jangwon Seo[1], Insoo Kim[1], Junhee Seok[1*]

[1]School of Electrical Engineering, Korea University, Seoul, Korea

[*] Correspondence should be addressed to jseok14@korea.ac.kr

Tables

Table S1. Comparison of R^2 performance for each axis of the six models: Interpolation, SRCNN, FSRCNN, VDSR, LapSRN, and FRSR in the training and validation sets. In the case of the training set, the VDSR model showed the highest learning performance, but the FRSR model showed the best performance in the validation set.

Table Data set		R^2					
		Interpolation	SRCNN	FSRCNN	VDSR	LapSRN	FRSR
Train	E_x	0.5253	0.9806	0.9775	0.9919	0.9853	0.9909
	E_y	0.8857	0.9831	0.9819	0.9938	0.9835	0.9930
	H_z	0.7707	0.9828	0.9804	0.9929	0.9873	0.9926
	average	0.7272	0.9821	0.9799	**0.9928**	0.9854	0.9921
Validation	E_x	0.5266	0.9790	0.9759	0.9850	0.9854	0.9897
	E_y	0.8842	0.9802	0.9807	0.9918	0.9837	0.9919
	H_z	0.7799	0.9818	0.9794	0.9916	0.9877	0.9919
	average	0.7303	0.9803	0.9787	0.9895	0.9856	**0.9912**

Table S2. Comparison of RMSE score for each axis of six models: Interpolation, SRCNN, FSRCNN, VDSR, LapSRN, and FRSR on the training and validation sets. In the case of the training set, the VDSR and FRSR models showed the highest learning performance with similar RMSE values, but the FRSR model showed the best performance on the validation set.

Data set		RMSE					
		Interpolation	SRCNN	FSRCNN	VDSR	LapSRN	FRSR
Train	E_x	0.0533	0.0056	0.0068	0.0038	0.0050	0.0038
	E_y	0.0122	0.0035	0.0041	0.0025	0.0035	0.0024
	H_z	0.0208	0.0051	0.0060	0.0034	0.0042	0.0034
	average	0.0288	0.0047	0.0056	**0.0032**	0.0042	**0.0032**
Validation	E_x	0.0532	0.0058	0.0074	0.0040	0.0051	0.0040
	E_y	0.0122	0.0036	0.0045	0.0027	0.0036	0.0025
	H_z	0.0208	0.0052	0.0066	0.0035	0.0044	0.0035
	average	0.0287	0.0049	0.0061	0.0034	0.0044	**0.0033**

Table S3. Comparison of R^2 performance for each axis of six models: Interpolation, SRCNN, FSRCNN, VDSR, LapSRN, and FRSR in the test set. FRSR showed the best performance for the R^2 score.

Data set		R^2					
		Interpolation	SRCNN	FSRCNN	VDSR	LapSRN	FRSR
Test	E_x	0.5151	0.9872	0.9791	0.9929	0.9915	0.9936
	E_y	0.9011	0.9903	0.9828	0.9948	0.9930	0.9945
	H_z	0.7850	0.9891	0.9818	0.9945	0.9926	0.9942
	average	0.7337	0.9889	0.9813	0.9940	0.9924	**0.9941**

Table S4. Comparison of RMSE score for each axis of the six models: Interpolation, SRCNN, FSRCNN, VDSR, LapSRN, and FRSR in the test set. FRSR showed the best performance in terms of the RMSE score.

Data set		RMSE					
		Interpolation	SRCNN	FSRCNN	VDSR	LapSRN	FRSR
Test	E_x	0.0536	0.0043	0.0064	0.0035	0.0039	0.0033
	E_y	0.0120	0.0028	0.0040	0.0023	0.0025	0.0022
	H_z	0.0207	0.0037	0.0056	0.0031	0.0035	0.0030
	average	0.0288	0.0036	0.0053	0.0030	0.0033	**0.0028**

Table S5. Comparison of evaluation metrics of the six models: Interpolation, SRCNN, FSRCNN, VDSR, LapSRN, and FRSR in the test set. In general, FRSR showed the best performance in terms of evaluation scores.

Evaluation Metrics	LR	Interpolation	SRCNN	FSRCNN	VDSR	LapSRN	FRSR
PSNR	20.72	25.38	48.65	41.79	50.80	49.11	**50.82**
SSIM	0.9765	0.9860	0.9995	0.9987	**0.9997**	0.9995	**0.9997**
VIF	0.3212	0.5681	0.9234	0.8198	**0.9592**	0.9103	0.9484
UQI	0.9789	0.9915	0.9999	0.9996	**1.000**	0.9999	**1.000**
RASE	326.3	190.0	26.15	57.18	16.68	22.94	**16.61**
SAM	0.1466	0.0858	0.0056	0.0128	**0.0044**	0.0054	**0.0044**
SCC	0.0693	0.1313	0.1513	0.1360	0.1565	0.1492	**0.1581**

Table S6. Comparison of power flux for each of the six models: Interpolation, SRCNN, FSRCNN, VDSR, LapSRN, and FRSR on the test data set.

Model	interpolation	SRCNN	FSRCNN	VDSR	LapSRN	FRSR
average of RMSE	0.0074	0.0041	0.0046	**0.0031**	0.0047	**0.0031**
average of R^2	0.9739	0.9911	0.9897	0.9942	0.9875	**0.9943**

Table S7. Network architecture of the Interpolation model used for training.

Interpolation Block		
Description	Up sampling	Output shape (W×H×C)
Input		200×200×3
Interpolation-image	×2	400×400×3

Table S8. Network architecture of the FRSR model used for training.

FRSR			
Description	Kernel size	Activation	Output shape (W×H×C)
Input			200×200×3
Conv2d_1	5×5	Leaky ReLU	200×200×64
Conv2d_2	1×1	Leaky ReLU	200×200×64
Conv2d_3~7	3×3	Leaky ReLU	200×200×64
Conv2d_8	1×1	Leaky ReLU	200×200×64
Deconv2d	9×9		400×400×3 (Residual output)
Residual output + Interpolation Block			400×400×3

Table S9. Network architecture of the SRCNN model used for training.

SRCNN			
Description	Kernel size	Activation	Output shape (W×H×C)
Input			200×200×3
Interpolation Block			400×400×3
Conv2d_1	9×9	ReLU	400×400×64
Conv2d_2	9×9	ReLU	400×400×32
Conv2d_3	5×5	Linear	400×400×3

Table S10. Network architecture of the FSRCNN model used for training.

FSRCNN			
Description	Kernel size	Activation	Output shape (W×H×C)
Input			200×200×3
Conv2d_1	5×5	PReLU	200×200×56
Conv2d_2	1×1	PReLU	200×200×16
Conv2d_3~6	3×3	PReLU	200×200×12
Conv2d_7	1×1	PReLU	200×200×56
Deconv2d	9×9		400×400×3

Table S11. Network architecture of the VDSR model used for training.

VDSR			
Description	Kernel size	Activation	Output shape (W×H×C)
Input			200×200×3
Interpolation Block			400×400×3
Conv2d_1~8	3×3	ReLU	400×400×64
Conv2d_9	3×3		400×400×3<br>(Residual output)
Residual output<br>+<br>Interpolation Block			400×400×3

Table S12. Network architecture of the LapSRN model used for training.

LapSRN			
Description	Kernel size	Activation	Output shape (W×H×C)
Input			200×200×3
Conv2d_1~4	3×3	Leaky ReLU	200×200×32<br>(embedding output)
Input			200×200×3
Conv2d_5~6	3×3	Leaky ReLU	200×200× 32
Deconv2d	4×4		400×400×3<br>(up-sampling output)
Input(embedding output)	3×3	Leaky ReLU	200×200×32
Conv2d_7~11	3×3	Leaky ReLU	200×200×32<br>(layer output)
embedding output<br>+<br>layer output			200×200×32
Deconv2d	4×4		400×400×3
Conv2d_12~14	3×3	Leaky ReLU	400×400×3<br>(Residual output)
up-sampling output<br>+<br>Residual output			400×400×3

Figure

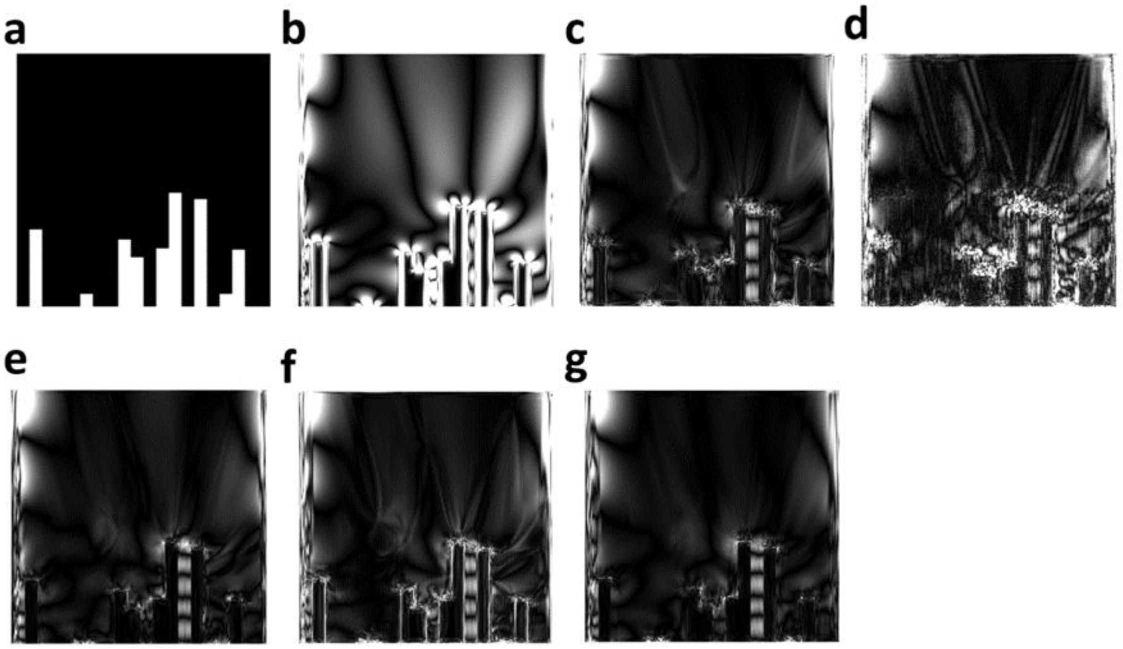

Figure S1. An example of a study showing the RMSE error between the predicted and actual values of the $\boldsymbol{E_x}$ component for a specific slit design at 800 nm wavelength as a heatmap. (a) Device design is used in the example. (b), (c), (d), (e), and (f) show black when the error is small and white when the error is large when a low-resolution image is restored to a high-resolution image. (b) Interpolation is applied, (c) SRCNN is applied, (d) FSRCNN is applied, (e) VDSR is applied, and (f) LapSRN is applied, and (g) FRSR is applied.
